# Evaluating tourniquet use in Swedish prehospital care for civilian extremity trauma

**DOI:** 10.1007/s00068-020-01341-0

**Published:** 2020-04-29

**Authors:** Eric Wellme, Victor Mill, Carl Montán

**Affiliations:** 1 Department of Medicine and Surgery, Karolinska Institutet, Stockholm, Sweden; 2 Department of Vascular Surgery, Karolinska University Hospital Solna, 171 76 Stockholm, Sweden

**Keywords:** Haemorrhage, Tourniquet, Extremity trauma, Prehospital, Bleeding, Civilian

## Abstract

**Purpose:**

The use of tourniquet (TQ) is today a well-documented and lifesaving adjunct to control bleeding from extremity trauma in the military setting. Since August 2015, the ambulance services in Stockholm, Sweden are equipped with TQs. The implementation and potential complications related to TQ use have so far not been evaluated. The primary aim of this study was to evaluate the prehospital use of TQ for haemorrhage control in extremity trauma. Possible complications following the use of TQ were analysed.

**Methods:**

A retrospective, descriptive cohort study of extremity haemorrhage for all patients (*n* = 56) with a documented prehospital use of TQ admitted to the trauma centre at Karolinska University Hospital from 1st August 2015 to 31st December 2017 was conducted. Data regarding TQ use including indication, duration, bleeding volume, complications and definitive injury were analysed.

**Results:**

Out of 63 placements of TQ in 56 patients, TQ stopped the bleeding effectively in 98.2% of the cases and the TQ time varied from 15 to 100 min. The overall complication rate was 30.1%; however, complications possibly related to TQ use were 3.6%. In 16 (28.6%) cases, the TQ were used for a non-life-threating haemorrhage which may have been stopped with direct pressure only.

**Conclusion:**

This study shows TQs to be an effective but overused tool in haemorrhage control. The use of TQ was not associated with any severe complications, implying the safety and effectiveness of the device in the civilian setting if TQ time is kept under 100 min.

## Introduction

Trauma is the most common cause of death in Sweden among people in the ages between 15 and 44 [1]. Uncontrolled haemorrhage is, after neurotrauma, the leading cause of death in civilian trauma. A study from 2014 [2], suggested that approximately 28% of all trauma patients had potentially survivable injuries if given the correct treatment. Out of the possibly preventable deaths, over 50% were caused by uncontrolled haemorrhage [2]. Rapid control and treatment of significant bleeding in trauma patients is, therefore, vital [3]. In the city region of Stockholm, tourniquets (TQs) (brand name CAT^®^) were introduced as a tool for prehospital ambulances in August 2015.

The majority of literature supporting the use of TQs is based on data from military conflicts where the use of TQs is associated with improved survival and a low complication rate in severe extremity trauma [4]. The use of TQ in civilian trauma has only recently been adopted, due to concerns of complications such as nerve damage, compartment syndrome, amputation, secondary vascular and tissue injuries [5, 6].

In Sweden, no official guidelines have been published for the civilian ambulance services regarding indications for TQ in trauma [7]. The primary aim of this study was to evaluate the prehospital use of TQ for haemorrhage control in extremity trauma and to analyse possible complications of TQ use and the conjunctive measures such as blood transfusion.

## Methods and materials

This was a retrospective, descriptive cohort study of all adult (> 16 years) extremity trauma patients with bleeding and documented use of TQ admitted to Karolinska University Hospital Stockholm, Sweden between 1st of August 2015 and 31st of December 2017. Patients were identified using ICD coding, corresponding to injuries where TQ might have been indicated. Indication for application of TQ was evaluated with regard to the final diagnosis (given in hospital) to assess if an overuse of TQ could be suspected in comparison to only using covering pressure bandage.

Patients’ data (including TQ time, physical parameters and laboratory data) were extracted from computerized prehospital and hospital medical records. In cases where data were missing, the prehospital medical records systems were used and vice versa. Patients were cross-checked with the local transfusion registry. Injury severity score (ISS) was retrieved from the local trauma registry at Karolinska University hospital.

Mortality and complications possibly related to TQ use (i.e. compartment syndrome, amputation, secondary nerve injuries and kidney injury) were analysed.

Results and outcome measures were analysed using IBM^®^ SPSS Statistics^®^ for Macintosh version 24.0 (Armonk, New York, USA).

Ethical permission for this study was given from the local Ethical Review Board in Stockholm, Sweden (Reference number 2018/200-31).

## Results

**Fig. 1:**
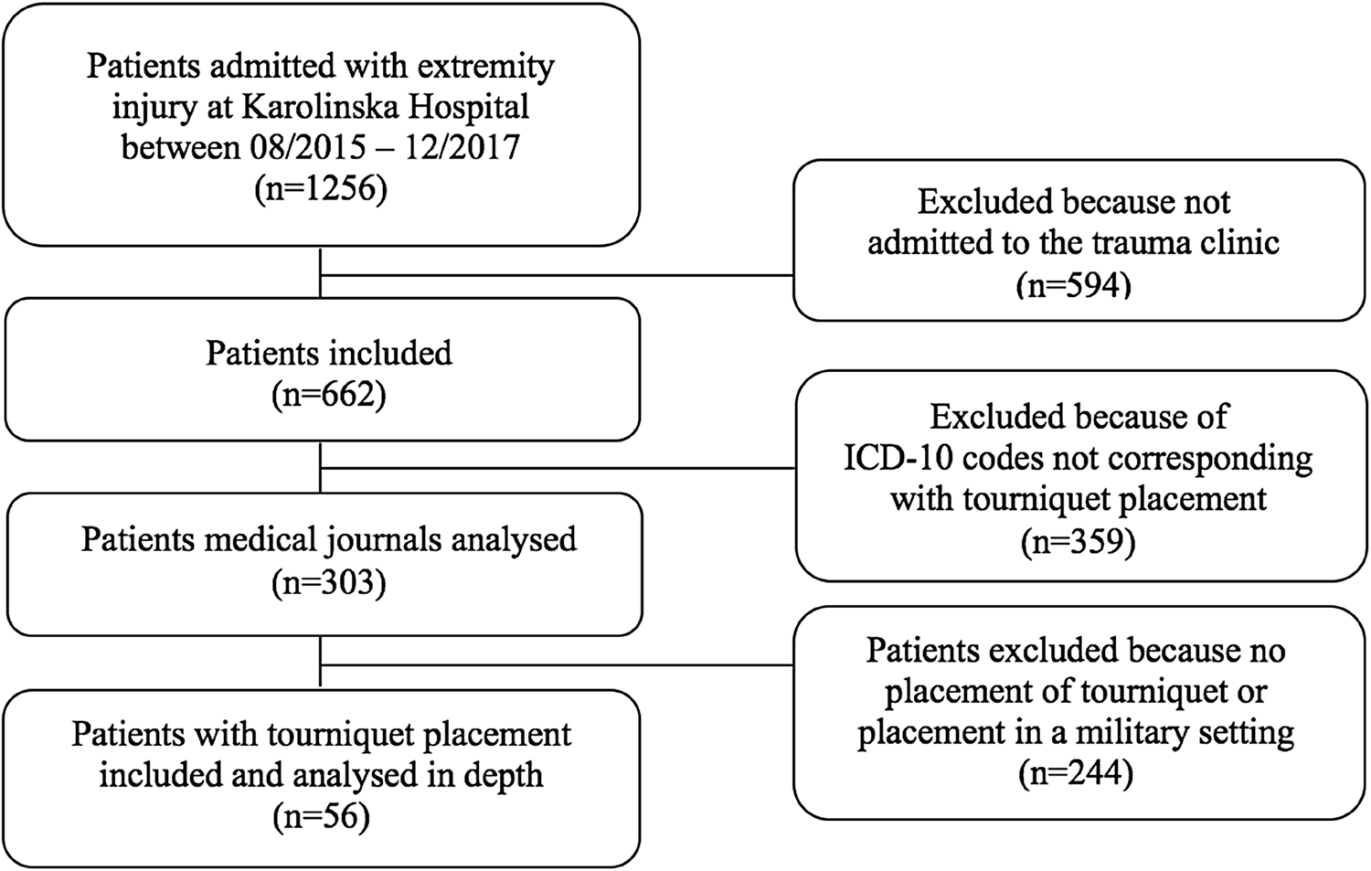
Flowchart describing how patients were identified for inclusion in study according to whether they received tourniquet or not. ICD-10 = International Statistical Classification of Diseases and Related Health Problems, 10th revision

**Fig. 2:**
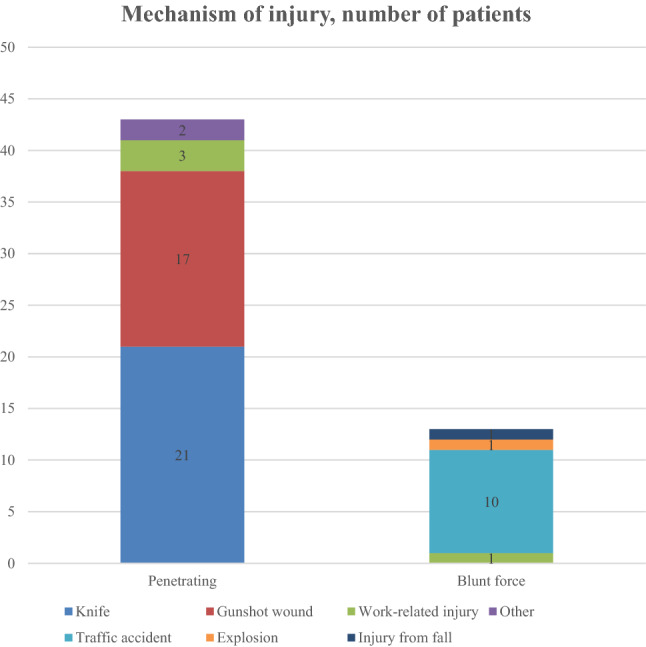
Mechanism of injury indicating use of tourniquet. 63 tourniquets in 56 patients

During the study period, 662 patients with extremity trauma were admitted to the trauma unit. Out of these, 303 patients’ admission notes and ambulance reports were analysed. Fifty-six patients with a documented prehospital placement of TQ were identified and included in the study (50 males and 6 females) (Table 1). Paediatric patients, burn trauma and military trauma were excluded. Flowchart for patient identification described in Fig. 1. 12 patients needed intensive care with a mean length of stay in the intensive care unit (ICU) of 7.58 days. Out of the 36 patients arriving with ambulance, 31 patients had a Glasgow Coma Scale (GCS) between 14 and 15 prior arriving to hospital, indicating that the majority of the patients were awake with minor risk of brain damage. The most frequent mechanism of injury was penetrating trauma caused by knives or firearms (Fig. 2). Among blunt trauma, the majority of injuries were caused by traffic accidents (Table 2; Fig. 2).

**Table 1:** Demographic and clinical parameters of patients with a placement of tourniquets

Demographics		*N* (range)
Age, median		31 (17–80)
Gender, men (%)		50 (89.3%)
Length of Stay, median		3 (1–62)
Days in ICU, median		0 (0–26)
Prehospital vital signs	Median (IQR)	Range
Pulse, beats/min (*n* = 38)	85 (70; 115)	(0–142)
Systolic blood pressure, mmHg (*n* = 33)	115 (100; 140)	(0–170)
Vital signs on admission	Median (IQR)	Range
Pulse, beats/min (*n* = 47)	90 (75; 100)	(0–150)
Systolic blood pressure, mmHg (*n* = 48)	122 (110; 140)	(0–180)
ISS, (*n* = 54)	9 (4; 16.25)	(1–75)
Blood transfusion, yes (%)	18 (32.1%)	
Massive blood transfusion, yes (%)	8 (14.3%)	
Labs on admission	Median (IQR)	Range
Haemoglobin, mg/l (*n* = 55)	138 (118; 146)	(82–168)
PK(INR) (*n* = 50)	1.1 (1.0; 1.1)	(0.9–2.7)
Fibrinogen, g/l (*n* = 49)	2.3 (1.8; 3.0)	(0.9–4.3)
Creatinine, micromole/l (*n* = 54)	90.5 (74.5; 101)	(51–131)
pH (*n* = 40)	7.36 (7.31; 7.39)	(6.8–7.56)
Lactate, mg/dl (*n* = 41)	2.6 (1.7; 4.8)	(0.7–13.7)

Contains information regarding *n* = 56 patients. Where indicated, not all patients were included

*ICU* intensive care unit, *ISS* Injury Severity Score, *IQR* interquartile range 25; 75 percentiles

**Table 2:** Mechanism of injury indicating use of tourniquet

Mechanism of injury	*N* (%)
Penetrating	43 (76.8)
Knife	21 (37.5)
Gunshot wound	17 (30.4)
Work-related injury	3 (5.4)
Other	2 (3.6)
Blunt force	13 (23.2)
Traffic accident	10 (17.9)
Explosion	1 (1.8)
Work-related injury	1 (1.8)
Injury from fall	1 (1.8)

63 tourniquets in 56 patients

The foremost indication (> 50%) for a placement of TQ was potentially life-threatening haemorrhage (Table 3). In several cases, a pulsating bleeding was present. Documented TQ time ranged from 15 to 100 min in 21 patients, in more than 50% of the cases data on TQ time was missing.

**Table 3:** The use of tourniquet

	*N* (%)
Indications	
Potentially life-threatening haemorrhage	27 (48.6)
Traumatic amputation	5 (8.9)
Open fracture	8 (14.3)
Non-life-threatening haemorrhage	16 (28.6)
TQ location	
Upper limb	10 (17.9)
Lower limb	46 (82.1)
TQ time, minutes (*n* = 21) median (IQR)	60 (30;60)
Patients with more than one TQ	7 (12.5)
Attempt with direct pressure before placement of TQ	
Yes	19 (33.9)
No	12 (21.4)
Unknown	25 (44.6)
Who placed the TQ	
Ambulance	27 (48.2)
Police	18 (32.1)
Emergency services	1 (1.8)
Bystanders	6 (11.5)
Unknown	4 (7.1)

Contains information regarding (*n* = 56) patients. Where indicated, not all patients were included

*TQ* tourniquet. *IQR* interquartile range 25; 75 percentiles

Based on medical records from the ambulance and the admission note, 16 (28.6%) of the injuries were categorised as non-life threatening. In four of these cases, the TQ was removed completely upon arrival at the scene by ambulance personnel, in all cases because of an unindicated use with no active bleeding.

In all but one patient, the TQ was sufficient to stop the bleeding effectively without the addition of a pressure bandage, making the TQ effective in 98.2% of the cases. No patient received two TQs on the same limb. In patients with a potentially life-threating haemorrhage, the TQ was applied after an attempt to stop the bleeding with direct pressure in more than 40% of the cases.

Almost one third (32.1%) of the patients had injuries requiring blood transfusion (Table 4). Eight of these received blood according to a massive transfusion protocol. In this group, four patients had a fibrinogen equal to or lower than 1.7 g/l; however, no correlation was found between low fibrinogen and blood transfusion. In one case, a blood pressure cuff was used as TQ and in nine cases, an improvised TQ was placed; most frequently a belt but also bed sheets and in one case, a dog leash. In four of these cases, the improvised TQ was replaced by a commercial TQ upon arrival of prehospital personnel.

**Table 4:** Specification of blood products for patients (*n* = 18) who received blood transfusion

Blood product	Number of units. mean (range)
Erythrocytes	11 (2–45)
Plasma	5.67 (0–22)
Platelets	1.22 (0–8)

Death occurred in two cases (30-day mortality 3.6%). Both patients arrived at the trauma unit with ongoing CPR. The first patient died in the ICU due to anoxic brain injury 2 days after admission following complications to massive haemorrhage from a stab wound in the femoral artery. The second patient was declared dead upon arrival to the trauma unit after multiple gunshot wounds to the chest and arms. The leading cause of death in both cases were exsanguination.

The total rate of complications was 30.1% (Table 5). There were seven amputations, five of which were traumatic due to the initial trauma. Two below knee amputations were necessary after admission to hospital, one due to post-operative infection and one following the initial trauma with development of necrotic ulcers. No amputations due to TQ use were seen in this study.

**Table 5:** Mortality and complications

	*N* (%)
30-day mortality	2 (3.6)
Total complications^a^	17 (30.1)
Amputations following admission to hospital	2
Fasciotomy	4
Prophylactic	3
Compartment syndrome	1
Acute kidney injury	3
Nerve damage^b^	13
Loss of motor function	10
Loss of sensory function	11
Other	1
No complications	39 (69.9)

Total frequency of mortality and complications related to either direct trauma or the use of tourniquet

^a^Four patients suffered more than one complication

^b^Eight patients had both loss of motor and sensory function

Four patients acquired a fasciotomy, of these three were made prophylactic. One case of compartment syndrome was identified. Three patients developed secondary acute kidney injury (AKI), one case due to fluid overload and pulmonary oedema, one due to septic shock and multi-organ failure, 7 days post-trauma and one due to hypoperfusion and rhabdomyolysis due to compartment syndrome. The latter two cases needed haemodialysis over a period.

Nerve damage was seen in 13 (23.2%) of the patients. In eight of these cases, the damage was clearly described to be caused by the initial trauma. In two cases, the nerve injury might have been caused by the application of TQ, according to the information provided in the medical records. In these cases, the nerve function recovered within 2 weeks. In three patients, it was not possible to verify the cause of the nerve damage; however, in these cases, the damage to the nerve was more distally than the site of the TQ. The nerve damage for 12 of the patients were transient within 2 weeks of the trauma. One patient with sensory nerve damage, due to the initial trauma, required 8 months until full recovery.

One patient developed a subcutaneous hematoma at the site of the TQ.

**Fig. 3:**
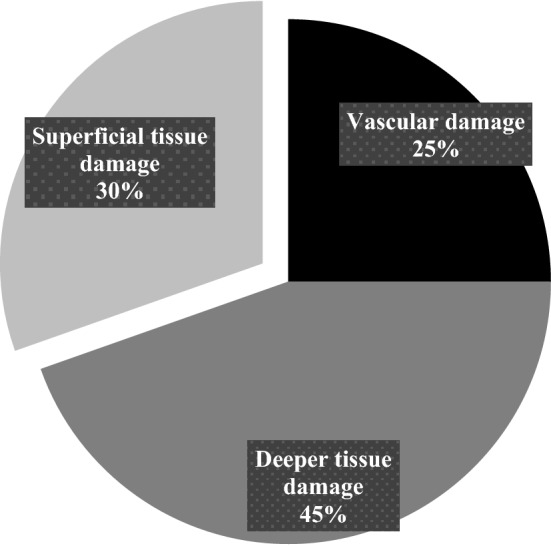
Patients categorized according to type of damage. Damages according to ICD-10 codes and intervention done after admission to hospital

All patients were categorized into three groups depending on the final diagnosis after admission—*vascular damage*, *deep tissue damage* and *superficial tissue damage* (Table 6; Fig. 3). All 14 (25%) patients in the first category needed vascular surgery or intervention. Patients in the second category (*n* = 25, 44.6%) required orthopaedic surgery, and wound revision. The 17 (30.4%) patients with a superficial tissue damage needed skin sutures, wound dressing or no intervention at all. The majority of patients in this group had an injury categorized as non-life threatening.

**Table 6:** Patients categorized according to type of damage

	*N* (%)
Vascular damage	14 (25.0)
Deeper tissue damage	25 (44.6)
Superficial tissue damage	17 (30.4)

Damages according to ICD-10 codes and intervention done after admission to hospital

## Discussion

This study provides the first evaluation of TQ use in Sweden since the formal introduction of the device in 2015. The results demonstrate an overuse based on the actual injury from the discharge note and the interventions performed at the trauma unit. Complications due to the use of TQ were low, two patients (3.6%) suffered transient nerve damage, it could not be determined if the cause was due to the use of a TQ, the initial trauma or a combination of both. Our results are consistent with three other studies from a military environment [8–10]. In a study by Lakstein et al. [10], five patients (5.5%) out of 91 suffered nerve damage attributed to the use of TQ, all affected patients had a TQ time over 100 min. Kragh et al. [8] reported a 1.7% complication rate in form of transient nerve damage at the site of TQ. Experiences from the United Kingdom military shows similar data where three complications were seen following over 100 TQ applications [9]. Most studies to date are based on military populations from conflict zones. However, recent studies have been conducted in a civilian environment [5, 11] with similar results. In contrast to previous fears, data indicate a low complications rate—at least with a TQ time below 100 min.

In the year of 1916, TQ was described as *“…the invention of the evil one”* [12] and in a study from 1962, it is stated that there is no place for TQ as an adjunct in acute bleeding [13]. As more recent studies have shown, the TQ is an effective and safe device in the initial care of the trauma patient, in both military [10] and civilian settings [14].

Our data indicate an overuse of TQ, as 30% of the applications were to non-life-threatening superficial tissue injuries. Bleeding from these injuries may have stopped by direct pressure only. This is a known phenomenon when handling an external bleeding, especially after gun violence [15]. In at least 20% of the cases, direct pressure was not tried prior to application of TQ. This indicates the need of an algorithm or guidelines for the use of TQ; guiding medical personnel in the field to a safe use and to minimize unindicated applications of the device.

However, none of the patients with superficial damages acquired any complication that could be related to TQ use, demonstrating that TQs can be used safely also for minor injuries. Some studies even promote an expanded use of TQ for the civilian trauma patient, stating that the risk of complications is low and that TQs are safe to use [4, 16, 17]. Cornelius et al. [18] seconds the claim and recommends the use of TQ, to minimize TQ time they advocate short transportation time to hospital.

None of the patients had a measured TQ time longer than 100 min. The reasons why no TQ time exceeded 2 h may be due to short transport times within Stockholm and early transport from the trauma scene. This is also seen in other studies conducted in a civilian environment [4].

In over 30% of the cases, police officers applied the TQ, mainly because they were the first responder on scene. No significant difference in complication rates was seen between TQs applied by police or ambulance personnel, a statement also observed in another study [19]. This demonstrates the benefit of all first responders, regardless of profession, being equipped with a TQ for a quick and easy tool in the initial care of a patient with extremity haemorrhage.

This study carries many limitations due to its retrospective nature. There are several cases of missing data, probably because the accumulated data have been collected from four different systems for registration (two registries and two computerized medical record systems).

A structured feedback and follow-up after introducing new devices and techniques to the Stockholm ambulance services seems to be lacking. This is the first evaluation of TQ since the implementation in 2015. To this date, no official guidelines for TQ use have been published for the ambulance services.

In conclusion, this study has proven TQs to be an effective but overused device, since the implementation of tourniquets for our ambulance services in 2015. The use of TQ was not associated with any severe complications implying both the benefit and safety of the device—if the TQ time is kept under 100 min. To minimize overuse, official guidelines for managing extremity bleeding could be helpful and improve safety for the civilian trauma patient.
